# Evaluation of product quality of low-fat sausage containing lotus rhizome root powders as affected by cooking method during storage

**DOI:** 10.5713/ab.25.0110

**Published:** 2025-07-11

**Authors:** Zhuangzhuang Qiu, Koo Bok Chin

**Affiliations:** 1 School of Public Health, Jining Medical University, Jining, China; 2 Department of Animal Science, Chonnam National University, Gwangju, Korea

**Keywords:** Antioxidant Activities, Ethanolic Extracts, Oven-dried Lotus Root Powder, Physicochemical Properties, Sausages, Shelf-life

## Abstract

**Objective:**

This study investigated the antioxidant activities of lotus root, *Nelumbo nucifera*, by producing oven-dried lotus root powder (ODLRP) and powdered ethanolic extracts of the ODLRP produced using different concentrations of ethanol (25%, 50%, 75%, and 100%) for various times (0–8 h). The effect of ODLRP and an ethanolic extract powder produced by an extraction of ODLRP with 50% ethanol for 4 h (ODLRP_E50%) in low-fat boiled and smoked pork sausages were investigated.

**Methods:**

Antioxidant activities of the ODLRP and the ethanol extracts produced from the ODLRP under different extraction conditions were analyzed. Then, the ODLRP and ODLRP_E50% extract powder were added to pork sausages at concentrations of 1% and 0.1%, respectively, to determine their effects on the physicochemical properties, and antibacterial and antioxidant activities the sausages.

**Results:**

The ODLRP ethanol extract powder exhibited antioxidant activity, regardless of extraction time. The pH value of the boiled pork sausages containing 1% ODLRP was lower than that of the control sausages (CS). The color value demonstrated that the lightness of the boiled and smoked sausages decreased with the addition of 1% ODLRP or 0.1% ODLRP_E50% compared with the CS, but their redness and yellowness increased. The hardness and gumminess of smoked sausages with 0.1% ODLRP_E50% were lower than those of the CS (p<0.05). However, the springiness of the boiled sausages containing 0.1% ODLRP_E50% decreased. Boiled sausages treated with ODLRP and ODLRP_E50% had lower thiobarbituric acid reactive substances compared with the CS from day 14 of storage. Moreover, the boiled sausages containing 1% sausages containing 0.1% ODLRP_E50% extract powder exhibited an extended shelf-life compared with the CS.

**Conclusion:**

Ethanol extraction conditions of ODLRP and cooking methods of low-fat pork sausages are critical factors affecting the quality and shelf-life of sausages.

## INTRODUCTION

Foods containing phenolic compounds have antioxidant activity, which can reduce the risk of diseases and improve health conditions in humans [1], including neurodegenerative and cardiovascular diseases, diabetes, and cancer [2]. Synthetic antioxidants can exhibit toxic effects or undesirable characteristics, causing concern among consumers and limiting their use in foods [1]. Therefore, consumers have a preference for natural antioxidants derived from vegetables or fruits. Consequently, researchers have focused on using natural antioxidants in the food and meat industries to extend products’ shelf life and improve their quality. For example, Kumar et al [3] reported that pineapple peel extract enhanced the antioxidant activity of polyvinyl alcohol–corn starch films due to its high 1,1-diphenyl-2-picrylhydrazyl (DPPH) radical scavenging ability, which could be useful in food packaging. In addition, grape seeds demonstrate antioxidant activity because they contain complex phenols. Their total phenolic content (TPC) and free radical scavenging activity have been found to significantly increase with heat treatment at 150°C for 40 min, thereby increasing their antioxidant activity [4]. In addition, Padda and Picha [5] assessed the antioxidant activities of sweet potatoes and reported that storage at a low temperature of 5°C for 2 weeks increased their antioxidant capacity by increasing the TPC.

Lotus root (LR), the edible rhizome of *Nelumbo nucifera*, has received increasing attention because of the presence of natural antioxidant phenolic compounds, such as gallic acid, catechin, and epicatechin [6]. The rhizome is a white aquatic vegetable that has an attractive aroma and is rich in nutrients; however, surface browning caused by the enzymatic oxidation of phenols can occur on fresh-cut LR, which can affect its consumption and acceptance [7]. In this study, LR powder (LRP) was prepared by oven drying at 100°C based on the findings of Qiu and Chin [8]; they reported that sausages containing 100°C oven-dried LRP (ODLRP) exhibited better resistance to lipid oxidation than sausages containing freeze-dried LRP. However, they reported that the physicochemical properties of regular-fat model sausages, such as their color and texture, increased with the addition of ODLRP, which may affect consumer acceptance.

Therefore, based on the hypothesis reported in existing study that extraction methods can increase LR’s functional properties (such as retaining functional substance for anti-obesity components), ethanolic extracts of ODLRP were prepared using different ethanol concentrations and extraction times to retain functional substances [9]. For instance, a hot water extract of taurine-added LR exhibited antioxidant and hepatoprotective effects in rats with high-fat-diet-induced obesity [10]. Therefore, based on the hypothesis that ethanolic extracts of ODLRP may have different antioxidant effects, this study investigated the antioxidant activities of ODLRP and ODLRP extracted using different concentrations of ethanol for different times in pork sausages. Then, ODLRP or ODLRP extract powder was added to boiled and smoked pork sausages to assess the effects on their physicochemical, antibacterial, and antioxidant properties.

## MATERIALS AND METHODS

### Preparation of oven-dried lotus root powder and its ethanolic extract powders

The LRs were chopped into slices (3 cm) and dried in a forced convection drying oven (LDO-250F; Daehan Labtech) at 100°C for 8 h. Then, the dried slices were pulverized into powder with a particle size of <150 μm and stored at −70°C. Subsequently, the ODLRP was extracted using different concentrations (25%, 50%, 75%, and 100%) of ethanol for various times (0, 2, 4, 6, and 8 h). The ODLR extracted with 50% ethanol for 4 h (ODLRP_E50%) was freeze-dried (FD5508; ilShinBioBase) for 24 h, blended into a powder, and stored at −70°C until use.

### Preparation of boiled and smoked pork sausages

Pork hams were purchased, trimmed, and ground using a meat grinder (M-12s; Fujee Cold & Machine). Three batches were prepared by mixing the meat batter with the different ingredients according to the formulations provided in Table 1. The meat batter was stuffed into 45-mm-diameter transparent polyvinylidene dichloride casings. Then, half of the stuffed sausages were boiled in a water bath (WB-22; Daihan Scientific) at 75°C for 30 min. The other half of the sausages were subjected to a smoking procedure, which consisted of reddening, drying, smoking, and steam cooking until the core temperature reached 72°C. After the boiled and smoked sausages were cooled to room temperature, they were peeled and vacuum-packed. Their physicochemical, antioxidant, and antimicrobial properties were assessed during storage at 10°C±1°C for 35 days.

### Analysis of the antioxidant activities of oven-dried lotus root powder and oven-dried lotus root powder_E50%

The ODLRP and its ethanolic extracts prepared at different concentrations of ethanol (0%, 25%, 50%, 75%, and 100%) were each dissolved in 15 mL of double-distilled water, and different concentration gradients (0%, 0.25%, 0.50%, 0.75%, and 1.00%) were obtained. The antioxidant activities of the prepared ODLRP and ethanolic extract solutions were measured by assessing their TPC, DPPH radical scavenging activity, ferrous iron chelating ability (FICA), and ferric reducing power ability (FRPA) according to the methods of Kim and Chin [11]. First, 1 mL of ODLRP or ethanolic extract solution was mixed with 0.25 mL of methanolic DPPH radical solution (0.2 mmol L^−1^). After leaving the mixture in the dark for 30 min, the DPPH radical scavenging activity was assessed at a wavelength of 517 nm in the ultraviolet spectral.

To assess the FICA, 0.5 mL of each solution was mixed with 100 μL of ferrous chloride (0.6 mmol L^−1^). Then, 1 mL of methanol or 0.9 mL of methanol with 100 μL of ferrozine (5 mmol L^−1^) was added to each mixture, and the mixture was allowed to stand at room temperature for 5 or 10 min, respectively, before measuring the absorbance at a wavelength of 562 nm in the ultraviolet spectral. For assessment of the FRPA, 2.5 mL of each solution was mixed with 2.5 mL (0.2 mmol L^−1^) sodium phosphate buffer (pH 6.6) and 10 mg mL^−1^ potassium ferricyanide. Each mixture was treated with 2.5 mL of 100 mg mL^−1^ trichloroacetic acid after incubation in a drying oven at 50°C for 20 min, and then centrifuged at 670×g for 10 min. Eventually, 2.5 mL of each supernatant was mixed with 2.5 mL of double-distilled water and 0.5 mL (1 mg mL^−1^) ferric chloride or 3 mL of double-distilled water, and then left for 10 min before measuring the absorbance at a wavelength of 700 nm in the ultraviolet spectral.

To assess the TPC, each 1% solution (100 μL) was mixed with 2.9 mL of double-distilled water, 2 mL of 2% sodium carbonate (Na_2_CO_3_), and 100 μL of 50% Folin–Ciocalteu reagent. Finally, each mixture was allowed to stand for 30 min, and the absorbance was measured at a wavelength of 750 nm.

### Analysis of the pH and color values of the boiled and smoked pork sausages

The pH value of the boiled or smoked sausages was measured five times using a pH meter (Mettler-Toledo International). The color values of each batch of boiled and smoked sausages were assessed by examining the lightness (L*), redness (a*), and yellowness (b*) using a color reader (Model CR-10; Konica Minolta).

### Texture profile analysis and determining the expressible moisture of the boiled and smoked pork sausages

The texture profile analysis (TPA) of each sausage was performed using the method of Caine et al [12] with slight modifications. Each sausage sample was cut into 10 small pillars, 1.3×1.3 cm. Then, each pillar was compressed using a 500 N load cell in an Instron Model 3344 testing system (Instron) at 300 mm/min.

For measuring the expressible moisture (EM) of the sausage samples, a 1.5 g square sample was cut from each sausage and wrapped in three-quarters of a filter paper. Then, the EM was calculated based on the difference in the weight of the filter paper before and after centrifugation at 1,500×g for 15 min using the formula as follows:


(1) EM (%)=ΔE×100/T

Where ΔE is the difference in the weight of the filter paper before and after centrifugation, and T is the initial weight of the sample.

### Proximate composition analysis of the boiled and smoked pork sausages

The protein, fat, and moisture content of a sample from each sausage was measured according to the methods of Brown and Zayas [13], with minor modifications. The protein content was determined using a Kjeldahl semi-automatic machine (#4002851; J.P. Selecta), while the fat content was determined by Soxhlet fat extraction. To determine the moisture content, a sample from each sausage was dried in an oven at 102°C for 16–24 h. The moisture content was calculated based on the difference in weight before and after drying.

### Analysis of the thiobarbituric acid reactive substances of the boiled and smoked pork sausages

The thiobarbituric acid reactive substances (TBARS) method of Sinnhuber and Yu [14] was used with slight modifications to assess lipid oxidation. In brief, 2 g of each sausage was treated with 3 mL of 2.5% trichloroacetic acid and 17 mL of 1% thiobarbituric acid. The mixture was boiled at 90°C for 30 min. Then, 5 mL of the supernatant was mixed with 5 mL of chloroform and centrifuged at 670×g for 5 min. Immediately thereafter, 3 mL of the supernatant was mixed with 3 mL of petroleum ether and again centrifuged at 670×g for 10 min. Finally, the absorbance of the bottom layer was measured at 532 nm using a spectrophotometer (X-1200; Human Corporation).

### Determining the microbial counts of the boiled and smoked pork sausages

A 10 g sample of each sausage was diluted with 90 mL of sterile water. Then, 0.1 mL of the mixture was evenly spread on total plate count and violet–red bile (VRB) agars and incubated at 37°C for 24 h. Finally, the number of colonies on the total plate count and VRB agar plates was recorded as the total bacterial count (TBC) and *Enterobacteriaceae* count (EBC), respectively, and expressed as log colony-forming units (cfu)/g.

### Statistical analysis

The TPCs of ODLRP and its ethanolic extracts were analyzed by one-way analysis of variance (ANOVA) using IBM SPSS Statistics (v.21.0 for Windows, IBM Statistical Software; IBM). The DPPH radical scavenging activity, FICA, and FRPA of the ODLRP and its ethanolic extracts were analyzed by two-way ANOVA, using concentration and treatment as the main factors. The main factors considered for the two-way ANOVA of each sausage sample were treatment and days of storage. Each experiment was repeated thrice, and each repetition was treated as a random effect. Significant differences were indicated using Duncan’s multiple range test at p<0.05.

## RESULT AND DISCUSSION

### Antioxidant activity of the ethanolic extracts of oven-dried lotus root powder

**Figure 1:**
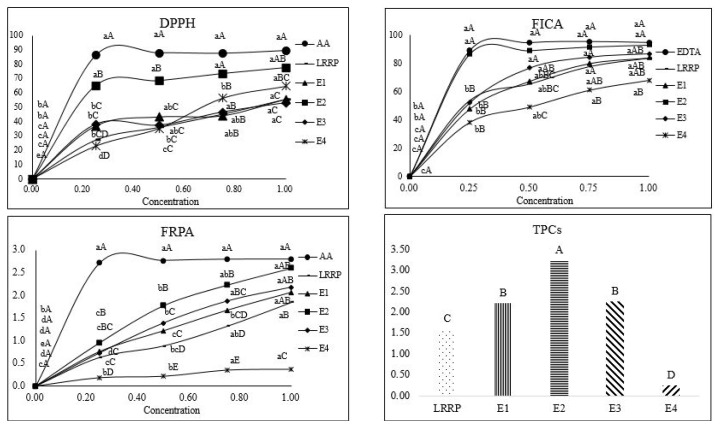
Antioxidant activity of ODLRP and ODLRP_E50% extracted with different concentrations of ethanol. ^A–E^ Mean with different superscripts in pooled data are different. ^a–e^ Mean with different superscripts are different (p<0.05). DPPH, 1,1-diphenyl-2-picrylhydrazyl; FICA, ferrous iron chelating ability; FRPA, ferric reducing power ability; TPC, total phenolic content; ODLRP, 100°C oven-dried lotus root powder; ODLRP_E50%, ODLRP extract (50% ethanol for 4 h) powder.

Among the ethanolic extracts of ODLRP, the extract obtained using 50% ethanol (ODLRP_E50%) had the highest TPC (Figure 1), while the lowest TPC was observed in the extract prepared with 100% ethanol (p<0.05). These results indicated that TPCs of the ethanolic extracts of ODLRP increased with increased ethanol concentration up to 50%, then declined with a further increase in ethanol concentration. This result is consistent with the results of Yang et al [15], who reported that crocin production increased with ethanol concentration up to 50.8%, followed by a decrease at ethanol concentrations up to 70%. They reported that the increased yield was due to increased damage to raw cell membranes caused by the higher concentrations of ethanol. Moreover, Özbek et al [16] reported that binary solvent systems extracted higher TPC than single solvent extractions. Therefore, ethanol and water at a ratio of 50:50 may be the highest possible extraction yield. This is supported by the results of this study, which demonstrated that the ODLRP_E50% had the highest TPC among all the ethanolic extracts of ODLRP.

The results of the DPPH radical scavenging activity were consistent with those of the TPC. Among all the ethanolic extracts, ODLRP_E50% had the highest DPPH radical scavenging activity, while ODLRP extracted with 100% ethanol (E4) showed the lowest (Figure 1). This result was supported by the findings of Özbek et al [16], who reported that DPPH radical scavenging activity was highly correlated with the TPC, showing a positive linear correlation. Yue et al [17] also assessed the effect of total polyphenols on the antioxidant activity, which was affected by the composition of polyphenols.

Between the concentration range of 50% to 75%, ODLRP extracted with 50% ethanol (E2) had the highest FICA, while E4 had the lowest FICA among all the ethanolic extracts of ODLRP (Figure 1). This finding is consistent with that of Kim and Chin [18], who reported that FICA could be affected by phenolic acids associated with phenolic compounds, as phenolic acids contain 3′,4′-dihydroxyl groups with potential iron-chelating capacity. Furthermore, the TPC was most affected by an ethanol and water ratio of 50:50, indicating that the antioxidant compounds in FICA were maximized when using binary solvents.

Within the ethanol range of 25%–75%, E2 had the highest FRPA, while E4 had the lowest among all the ethanolic extract powders (Figure 1). The FRPA can serve as an important indicator in Fe^3+^ to Fe^2+^ reduction assays, as it can be reacted with electron donors. Furthermore, Senevirathne et al [19] suggest that hydrophilic phenolic compounds increase the FRPA.

### The total phenolic content, 1,1-diphenyl-2-picrylhydrazyl radical scavenging activity, ferrous iron chelating ability, and ferric reducing power ability of oven-dried lotus root powder extract powders prepared using 50% ethanol for different extraction times

**Figure 2:**
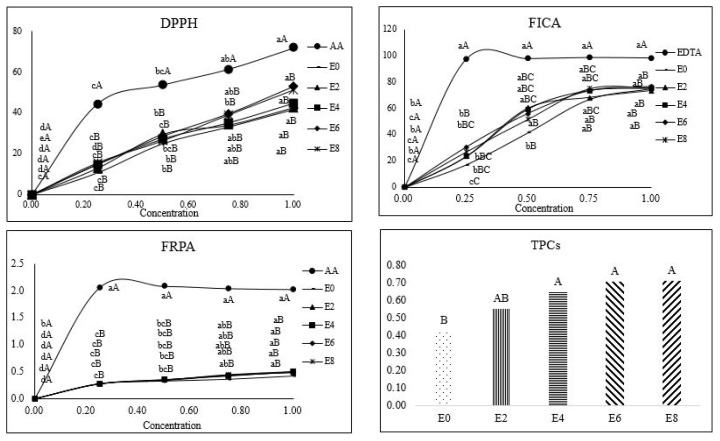
Antioxidant activity of ODLRP extracted with 50% ethanol at different times. ^A,B^ Mean with different superscripts In pooled data are different (p<0.05). ^a–d^ Mean with different superscripts are different (p<0.05). DPPH, 1,1-diphenyl-2-picrylhydrazyl; FICA, ferrous iron chelating ability; FRPA, ferric reducing power ability; TPC, total phenolic content; ODLRP, 100°C oven-dried lotus root powder.

Figure 2 shows the antioxidant activity of the extracts of ODLRP prepared using 50% ethanol for different extraction times (0–8 h). The results show that the antioxidant activity of the ethanolic extracts of ODLRP was affected by the ethanol concentration and extraction time. However, the ethanolic extracts of ODLRP extracted using 50% ethanol for 4, 6, and 8 h showed similar TPCs (Figure 1). This finding was supported by Yang et al [15], who reported that extraction time had a positive effect on the yield of geniposide. However, these authors reported that extraction after 35 min had minimal effect on the yield because the cell membranes were destroyed by the ethanol, affecting the release of TPC.

The DPPH radical scavenging activity and FRPA of the ODLRP extracted using 50% ethanol were not affected by the extraction time. The FICA of ODLRP extracted with 50% ethanol by 4 h (E4h = ODLRP_E50%) was higher than that extracted for 0 h at a concentration of 0.25% (p<0.05). However, no difference was noted among the other ethanolic extracts at different extraction times. These results may be due to the higher TPCs in E4h compared with ODLRP extracted with 50% ethanol by 0 h.

### Physicochemical, textural properties, and antioxidant and antimicrobial activity of boiled and smoked sausages containing 1% oven-dried lotus root powder or 0.1% oven-dried lotus root powder_E50%

Table 2 shows the pH and color values of boiled and smoked sausages. The pH values of boiled sausages treated with 1% ODLRP were lower than those of control sausage (CS) (p<0.05), likely due to the inherently low pH of the ODLRP (pH 5.74). The extraction rate of ODLRP_E50% was only 8.34%; therefore, the addition of 0.1% was unlikely to reduce the pH. During storage, the pH values of all the sausages tended to increase regardless of the treatment, possibly because the spoilage of sausage proteins resulted in the release of amines [20]. Furthermore, Kim et al [21] suggest that an increase in pH during storage may result from the accumulation of nonprotein nitrogen and amino acid catabolites.

Table 2 shows the pH and color changes of the boiled and smoked sausages manufactured with 1% ODLRP or 0.1% ODLRP_50%. When the color of the boiled sausages containing 1% ODLRP was compared with the CS, the L* value was lower, but the a* value increased. The boiled sausages containing 0.1% ODLRP_E50% also showed a decrease in L*, but the a* and b* values increased. The color changes in the sausages were attributed to the color of the added powders, since both ODLRP and ODLRP_E50% were brown–red. This study’s results were consistent with those of Qiu and Chin [8], who studied regular-fat model sausages supplemented with ODLRP and noted a decrease in L*, but an increase in a* and b* due to the reddish-yellow color of ODLRP.

In smoked sausages, the pH values did not change with the addition of 1% ODLRP or 0.1% ODLRP_E50% (p>0.05) (Table 2). However, the pH values decreased after 7 days of storage (p<0.05). This can be explained by the finding of Zhou et al [22], who reported that the pH values of smoked sausages decreased during storage because of microbial decomposition or rosemary components, which led to increased acid production. In addition, Yi et al [6] reported that adding ODLRP or ODLRP_E50% into smoked sausages led to the decomposition and release of phenolic acids, resulting in a reduction of the pH. The color change in smoked sausages showed the same trend as those in boiled sausages, being mainly affected by the color of the added ODLRP or ODLR_E50%. Compared with the CS, the L* of the smoked sausages containing 1% ODLRP or 0.1% ODLRP_C50% decreased, but the a* and b* increased. Moreover, the addition of 1% ODLRP resulted in a greater increase in b* compared with the addition of 0.1% ODLRP_E50% because of the higher quantity added.

Table 3 shows the results of TPA of boiled and smoked sausages with the addition of 1% ODLRP or 0.1% ODLRP_E50%. In the boiled sausages, the TPA results did not differ with the addition of the ODLRP or ODLRP_E50% (p<0.05), except for the springiness. However, Qiu and Chin [8] reported that adding 1% ODLRP or freeze-dried LRP increased the TPA parameter values in regular-fat model sausages and suggested that this increase was caused by the LRP’s dietary fibers. Moreover, Lee et al [23] reported that breakfast sausages treated with 2% dried kimchi powder showed lower springiness than CS because the kimchi powder’s insoluble fibers of affected the water binding capacity or swelling properties. In this study, the hardness and gumminess of smoked sausages decreased with the addition of 0.1% ODLRP_E50%. This result is consistent with that of Zhou et al [22], who reported that the addition of rosemary extract at concentrations higher than 0.1% decreased the hardness and gumminess of smoked sausages. Rosemary extract contains phenolic diterpenes, carnosic acid, and carnosol substances, which can react with proteins or increase water retention, thus decreasing the texture of smoked sausages. However, Ham et al [24] reported no differences in hardness and gumminess in cooked emulsion sausages containing 1%–3% LRP (p>0.05). The differences in TPA results between the two studies may be explained by the differences in sausage processing procedures, as smoking may affect LRP’s dietary fibers, thereby reducing the sausages’ hardness and gumminess.

The results of the proximate composition analysis and EM of the boiled and smoked sausages did not differ from the CS with the addition of 1% ODLRP or 0.1% ODLRP_E50% (Table 4), except for a few cases. The small differences observed in the sausages’ protein content may be explained by the ODLPR protein content, which was 11.28% in this study. However, the protein content of the boiled and smoked sausages decreased on day 35 of storage, but the changes were minor and considered negligible. These findings are aligned with those of Ham et al [24], who reported that the addition of between 1% and 3% LRP (protein content 7.84%) did not significantly affect the protein content of boiled and smoked sausages.

**Figure 3:**
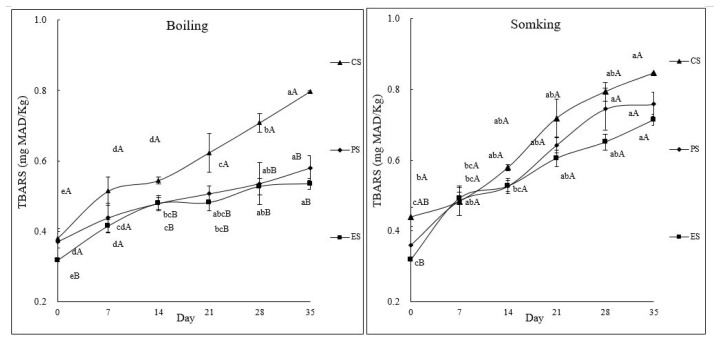
TBARS values of boiling and smoking sausages affected by the addition of ODLRP and ODLRP_E50%. ^A,B^ Mean with different superscripts In pooled data are different. ^a–e^ Mean with different superscripts are different (p<0.05). TBARS, thiobarbituric acid reactive substances; ODLRP, 100°C oven-dried lotus root powder; ODLRP_E50%, ODLRP extract (50% ethanol for 4 h) powder.

The TBARS levels of boiled sausages treated with 1% ODLRP or 0.1% ODLRP_E50% were lower than those of CS during storage (p<0.05) (Figure 3), indicating reduced lipid oxidation. This finding is supported by Qiu and Chin [8], who reported that the addition of ODLRP to sausages reduced their TBARS levels and retarded lipid oxidation, possibly because of the increased TPC. The ODLRP_E50% demonstrated the highest TPC and DPPH radical scavenging activity among the ethanolic extracts of all ODLRP (Figure 1); therefore, its addition to the sausages was anticipated to reduce the TBARS levels, indicating reduced lipid oxidation. However, the TBARS levels of smoked sausages treated with 1% ODLRP or 0.1% ODLRP_E50% did not differ from those of CS. This result may be due to the smoking process, which dehydrated the sausages and produced more phenolic and antioxidant compounds, such as monoethylene glycol and dimethyl ether, thereby decreasing lipid oxidation of the smoked sausages [25]. In addition, Schwert et al [26] have reported that the smoking process is a promising and safe method to manufacture Calabrese sausages with reduced protein contents and lipid oxidation. This study’s results support that the addition of 1% ODLRP or 0.1% ODLRP_E50% to sausages that are smoked results in lower lipid oxidation compared with control and boiled sausages.

**Figure 4:**
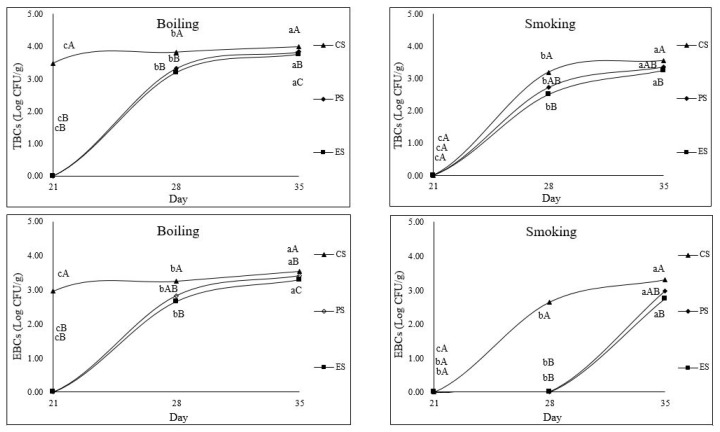
TBCs and EBCs of boiling and smoking sausages affected by the addition of ODLRP and ODLRP_E50%. ^A–C^ Mean with different superscripts In pooled data are different (p<0.05). ^a–c^ Mean with different superscripts are different (p<0.05). TBC, total bacterial count; EBC, *Enterobacteriaceae* count.

The TBCs and EBCs of boiled and smoked sausages are shown in Figure 4. The CS’ TBCs were detected from day 21 of storage. In contrast, the TBCs of the boiled sausages treated with either 1% ODLRP or 0.1% ODLRP_E50% were first observed on day 28 of storage (p<0.05), indicating slower microbial growth compared with the CS. At storage day 35, the TBCs of the boiled sausages treated with either 1% ODLRP or 0.1% ODLRPE were lower than those of the CS; however, the TBCs of the boiled sausages treated with ODLRP_E50% decreased to a greater extent, indicating that it had better antimicrobial activity than the ODLRP. The reduction in the sausages’ TBSs could be due to increased TPC levels resulting from the addition of either ODLRP or ODLRP_E50%. Lima et al [27] reported that phenolic compounds exhibited beneficial biological properties, e.g., their aromatic structure and the chemistry of multiple hydroxyl groups that can donate electrons or hydrogen atoms to neutralize free radicals and other reactive oxygen species.

The *Enterobacteriaceae* family consists of many bacteria, including *Salmonella* species, *Escherichia coli*, and *Shigella* species, which can grow in the gastrointestinal tract and harm human health [28]. The EBCs of boiled sausages were detected from day 28 of storage and were lower in sausages containing 0.1% ODLRP_E50% compared with the CS. On day 35 of storage, EBCs of boiled sausages treated with either 1% ODLRP or 0.1% ODLRP_E50% were lower than those of the CS; however, the EBCs of boiled sausages treated with ODLRP_E50% decreased to a greater extent. Therefore, the changes in the EBCs and TBCs in boiled sausages over time were similar. Moreover, the ODLRP and ODLRP_E50% exhibited antibacterial effects against *Enterobacteriaceae*, with the ODLRP_E50% exhibiting a better effect than the ODLRP. Qiu and Chin [8] reported that the addition of ODLRP to regular-fat sausages reduced the EBCs and adding ODLRP was more effective than adding freeze-dried LRP, as the ODLRP contained a higher TPC. The solubility of polyphenols depends on the hydroxyl groups, molecular size, and length of hydrocarbons, and a mixture of alcohol and water would be beneficial in adjusting the polarity of the alcohol solvent [29]. Consequently, in this study, the addition of 0.1% ODLRP_E50%, which contained higher TPCs, exhibited better antimicrobial effects in sausages compared with the addition of 1% ODLRP.

In the smoked sausages, TBCs were detected on day 28 of storage. However, only sausages containing 0.1% ODLRP_E50% had lower TBCs compared with the CS on day 35 of storage. In contrast, the EBC was first detected in the CS on day 28 of storage. At day 35 of storage, the EBCs in the smoked sausages containing 1% ODLRP or 0.1% ODLRP_E50% were lower than those in the CS. Therefore, the detection of microorganism growth in smoked sausages was delayed by a week in comparison with the growth in boiled sausages. Lingbeck et al [30] reported that during smoking, the pyrolysis of wood produces varying levels of phenols, carbonyls, and organic acids; this could have affected the growth of microorganisms in the smoked sausages, resulting in the extension of their shelf-life.

## CONCLUSION

The TPC of the ethanolic extract ODLRP_E50% was the highest among all the ethanolic extracts. Additionally, the 50% ethanol extract had the highest DPPH radical scavenging activity in the concentration range of 0.25%–0.75%, the highest FICA at 0.25%, and the highest FRPA at 0.50%. No differences in the TPC of ODLRP extracted with 50% ethanol were observed among the samples with extraction times of 4, 6, and 8 h. Similarly, the antioxidant activity did not differ at different extraction times, except that the FICA of the sample extracted for 6 h was higher than that of unextracted control sample.

The incorporation of 1% ODLRP into boiled sausages lowered their pH. However, the color values of both boiled and smoked sausages increased, regardless of the addition of 1% ODLRP or 0.1% ODLRP_E50%. In contrast, the addition of 1% ODLRP or 0.1% ODLRP_E50% decreased the TBARS levels of boiled sausages; however, no such effect was noted in smoked sausages. Adding 1% ODLRP or 0.1% ODLRP_E50% to the sausages reduced the microbial counts in boiled sausages, while adding 0.1% ODLRP_E50% inhibited microbial growth in smoked sausages. The microorganisms present in smoked sausages were detected one week later compared with boiled sausages. In conclusion, using 1% ODLRP or 0.1% ODLRP_E50% can extend the shelf-life of boiled sausages, but only 0.1% ODLRP_E50% effectively increases the shelf-life of smoked sausages, possibly due to its higher antimicrobial activity.

## Figures and Tables

**Table 1:** Low-fat pork sausage formulations treated with 1% ODLRP and 0.1% ODLRP_E50%

	Control		Sausage with 1% ODLRP powder		Sausage with 0.1% ODLRP_E50% extract powder	

	(%)	(g)	(%)	(g)	(%)	(g)
Raw meat	60.0	1,080.0	60.0	1,080.0	60.0	1,080.0
Water	31.53	567.5	31.53	567.5	31.53	567.5
Ice water	18.03	324.5	18.03	324.5	18.03	324.5
Hydrate water (soy protein isolate)	4.50	81.0	4.50	81.0	4.50	81.0
Hydrate water (konjac carrageenan)	9.0	162.0	9.0	162.0	9.0	162.0
Non-meat ingredient	8.47	152.5	9.47	170.5	8.57	154.3
Salt	1.28	23.0	1.28	23.0	1.28	23.0
Sodium tripolyphosphate	0.40	7.20	0.40	7.20	0.40	7.20
Prague powder	0.24	4.32	0.24	4.32	0.24	4.32
Sodium erythorbate	0.05	0.90	0.05	0.90	0.05	0.90
Sugar	1.00	18.00	1.00	18.00	1.00	18.00
Corn syrup	1.00	18.00	1.00	18.00	1.00	18.00
Spices	1.00	18.00	1.00	18.00	1.00	18.00
Whole-fat milk	1.00	18.00	1.00	18.00	1.00	18.00
Soy protein isolate	1.50	27.00	1.50	27.00	1.50	27.00
Konjac carrageenan	1.00	18.00	1.00	18.00	1.00	18.00
ODLRP	-	-	1.00	18.00	-	-
ODLR_E50% extract powder	-	-	-	-	0.10	1.80
Total	100.0	1,800.00	101.00	1,818.00	100.10	1,881.80

ODLRP, 100°C oven-dried lotus root powder; ODLRP_E50%, ODLRP extract (50% ethanol for 4 h) powder.

**Table 2:** pH and color values of boiled and smoked sausages as affected by the addition of 1% ODLRP and 0.1% ODLRP_E50%

Treatments	Boiled sausages				Smoked sausages			

	pH	L*	a*	b*	pH	L*	a*	b*
CS	6.23±0.06^a^	67.12±0.49^a^	8.72±0.20^b^	10.40±0.17^c^	5.78±0.11^a^	63.62±1.16^a^	8.71±0.33^b^	10.91±0.63^c^
PS	6.12±0.11^b^	65.43±0.28^c^	8.86±0.21^b^	12.02±0.22^a^	5.78±0.13^a^	62.26±0.89^b^	9.09±0.23^a^	12.63±0.43^a^
ES	6.18±0.07^ab^	65.85±0.40^b^	9.07±0.26^a^	10.88±0.22^b^	5.75±0.10^a^	62.37±0.49^b^	9.23±0.31^a^	11.33±0.47^b^
Storage days								
0	6.10±0.10^C^	66.37±0.92^A^	9.03±0.16^A^	11.10±0.73^A^	5.91±0.12^A^	63.33±1.13^A^	8.78±0.23^B^	11.03±0.99^C^
7	6.12±0.07^BC^	66.15±0.76^A^	8.97±0.22^AB^	11.13±0.77^A^	5.72±0.06^B^	62.72±1.11^A^	9.05±0.31^AB^	11.63±0.87^AB^
14	6.16±0.06^ABC^	66.10±0.80^A^	8.98±0.28^AB^	11.00±0.72^A^	5.68±0.06^B^	62.77±0.96^A^	8.98±0.40^AB^	11.35±0.77^BC^
21	6.21±0.09^AB^	66.00±1.13^A^	8.68±0.26^C^	11.18±0.97^A^	5.70±0.12^B^	62.43±1.20^A^	8.87±0.36^AB^	12.08±0.95^A^
28	6.24±0.07^A^	66.13±0.85^A^	8.90±0.29^ABC^	11.03±0.71^A^	5.79±0.05^AB^	62.73±1.09^A^	9.15±0.34^AB^	11.65±0.83^AB^
35	6.24±0.07^A^	66.03±0.77^A^	8.72±0.22^BC^	11.13±0.70^A^	5.81±0.06^AB^	62.50±1.15^A^	9.22±0.44^A^	12.00±0.88^A^

a–c Mean with different superscripts are different (p<0.05).

A–C Mean with different superscripts In pooled data are different (p<0.05).

ODLRP, 100°C oven-dried lotus root powder; ODLRP_E50%, ODLRP extract (50% ethanol for 4 h) powder; L*, lightness value; a*, redness value; b*, yellowness value; CS, control sausages; PS, sausages with 1% ODLRP; ES, sausages with 0.1% extract of ODLRP (50% ethanol for 4 h) powder.

**Table 3:** Texture profile analysis of boiled and smoked sausages affected by the addition of ODLRP and ODLRP_E50%

Treatment	Boiled sausage					Smoked sausage				

	Har	Spr	Gum	Che	Coh	Har	Spr	Gum	Che	Coh
CS	5,200±421^a^	7.32±0.17^ab^	42.0±10.3^a^	335±86.8^a^	9.22±0.79^a^	10,097±1,278^a^	7.32±0.23^a^	134±5.7^ab^	925±89^a^	12.63±0.46^b^
PS	5,240±551^a^	7.37±0.15^a^	43.6±10.3^a^	346±91.6^a^	9.60±1.06^a^	9,463±714^ab^	7.16±0.20^a^	139±12.4^a^	932±18^a^	13.02±0.49^a^
ES	5,073±536^a^	7.20±0.22^b^	42.6±9.4^a^	334±88.9^a^	9.06±0.32^a^	9,137±577^b^	7.16±0.23^a^	125±18.1^b^	888±44^a^	12.45±0.63^b^
Storage days										
0	4,770±246^A^	7.24±0.28^AB^	41.5±11.3^A^	333±87.9^A^	9.41±0.28^A^	8,869±456^A^	7.17±0.19^A^	133±9.0^A^	905±63^A^	12.73±0.59^AB^
7	4,927±528^A^	7.19±0.22^B^	41.6±10.2^A^	328±98.4^A^	8.80±0.86^A^	9,195±228^A^	7.25±0.32^A^	132±7.5^A^	921±57^A^	13.39±0.42^A^
14	5,183±722^A^	7.20±0.12^B^	42.6±8.9^A^	336±102.9^A^	9.16±1.69^A^	9,770±840^A^	7.10±0.21^A^	141±10.6^A^	932±74^A^	12.68±0.44^AB^
21	5,283±475^A^	7.39±0.12^AB^	43.8±10.6^A^	347±86.2^A^	9.45±0.35^A^	9,897±1383^A^	7.26±0.22^A^	126±19.6^A^	922±66^A^	12.21±0.39^C^
28	5,486±234^A^	7.35±0.18^AB^	42.4±11.8^A^	334±101.8^A^	9.51±0.33^A^	9,817±829^A^	7.11±0.23^A^	139±9.3^A^	900±47^A^	12.81±0.25^B^
35	5,378±367^A^	7.42±0.12^A^	44.4±9.8^A^	353±79.3^A^	9.43±0.30^A^	9,845±1387^A^	7.39±0.14^A^	126±20.9^A^	912±70^A^	12.38±0.60^AB^

a,b Mean with different superscripts are different (p<0.05).

A–C Mean with different superscripts In pooled data are different (p<0.05).

ODLRP, 100°C oven-dried lotus root powder; ODLRP_E50%, ODLRP extract (50% ethanol for 4 h) powder; CS, control sausages; PS, sausages containing 1% ODLRP; ES, sausages containing 0.1% extract of ODLRP (50% ethanol for 4 h) powder; Har, hardness (Kg); Spr, springiness (mm); Gum, gumminess (kg); Che, chewiness (kg×mm); Coh, cohesiveness (ratio).

**Table 4:** Proximate composition and expressible moisture (EM) of boiled and smoked sausages affected by the addition of ODLRP and ODLRP_E50%

Treatment	Boiled sausage				Smoked sausage			

	EM (%)	Protein (%)	Fat (%)	Moisture (%)	EM (%)	Protein (%)	Fat (%)	Moisture (%)
CS	16.09±0.47^a^	17.28±0.35^b^	1.44±0.17^a^	77.52±0.23^a^	13.06±0.89^a^	22.42±0.50^a^	2.18±0.14^a^	69.87±0.66^a^
PS	15.76±0.50^a^	17.43±0.29^ab^	1.40±0.15^a^	76.92±0.55^a^	13.14±0.52^a^	22.47±0.65^a^	2.21±0.28^a^	69.12±0.90^a^
ES	16.01±0.64^a^	17.57±0.12^a^	1.39±0.18^a^	77.17±0.88^a^	12.94±0.94^a^	22.65±0.37^a^	2.26±0.22^a^	69.63±1.22^a^
DAY								
0	15.78±0.42^A^	17.58±0.17^A^	1.43±0.17^A^	77.13±0.70^A^	13.28±0.97^A^	22.88±0.12^A^	2.18±0.21^A^	69.82±0.75^A^
7	16.18±0.51^A^	-	-	-	13.59±0.73^A^	-	-	-
14	15.67±0.70^A^	17.50±0.21^A^	1.40±0.13^A^	77.22±0.67^A^	12.61±0.82^A^	22.72±0.27^A^	2.26±0.23^A^	69.60±0.86^A^
21	16.26±0.28^A^	-	-	-	13.02±0.65^A^	-	-	-
28	15.93±0.60^A^	-	-	-	12.69±0.88^A^	-	-	-
35	15.91±0.64^A^	17.20±0.32^B^	1.39±0.19^A^	77.25±0.63^A^	13.10±0.42^A^	21.93±0.37^B^	2.22±0.23^A^	69.20±1.25^A^

a,b Mean with different superscripts are different (p<0.05).

A Means with common superscript In pooled data is not different (p>0.05).

ODLRP, 100°C oven-dried lotus root powder; ODLRP_E50%, ODLRP extract (50% ethanol for 4 h) powder; CS, control sausages; PS, sausages with 1% ODLRP; ES, sausages with 0.1% extract of ODLRP (50% ethanol for 4 h) powder.
